# Stakeholder perspectives on non-invasive brain stimulation

**DOI:** 10.1038/s41598-024-79118-3

**Published:** 2024-11-19

**Authors:** Moritz Julian Maier, Perianen Ramasawmy, Johannes Breuer, Anne Bansen, Antonio Oliviero, Georg Northoff, Andrea Antal

**Affiliations:** 1 Center for Responsible Research and Innovation at the Fraunhofer IAO, Berlin, Germany; 2 Non-Invasive Brain Stimulation Lab, Department of Neurology, University Medical Center Göttingen, Göttingen, Germany; 3 Bauhaus Universität Weimar, Weimar, Germany; 4 FENNSI Group, Hospital Nacional de Parapléjicos, SESCAM, Toledo, Spain; 5 Center for Clinical Neuroscience, Hospital Los Madroños, Brunete, Madrid Spain; 6 Mind, Brain Imaging and Neuroethics Research Unit, The Royal’s Institute of Mental Health Research, University of Ottawa, Ottawa, Canada

**Keywords:** Stakeholder engagement, Non-invasive brain stimulation (NIBS), Transcranial direct current stimulation (tDCS), Transcranial magnetic stimulation (TMS), Participatory process, Psychiatric disorders, Human behaviour, Medical ethics, Health policy

## Abstract

Non-invasive brain stimulation (NIBS) techniques such as transcranial direct current stimulation (tDCS) or transcranial magnetic stimulation (TMS) have made great progress in recent years and offer boundless potential for the neuroscientific research and treatment of disorders. However, the possible use of NIBS devices for neuro-doping and neuroenhancement in healthy individuals and the military are poorly regulated. The great potentials and diverse applications can have an impact on the future development of the technology and society. This participatory study therefore aims to summarize the perspectives of different stakeholder groups with the help of qualitative workshops. Nine qualitative on-site and virtual workshops were conducted in the study with 91 individuals from seven stakeholder groups: patients, students, do-it-yourself home users of tDCS, clinical practitioners, industry representatives, philosophers, and policy experts. The co-creative and design-based workshops were tailored to each group to document the wishes, fears, and general comments of the participants. The outlooks from each group were collected in written form and summarized into different categories. The result is a comprehensive overview of the different aspects that need to be considered in the field of NIBS. For example, several groups expressed the wish for home-based tDCS under medical supervision as a potential therapeutic intervention and discussed the associated technical specifications. Other topics that were addressed were performance enhancement for certain professional groups, training requirements for practitioners, and questions of agency, among others. This qualitative participatory research highlights the potential of tDCS and repetitive TMS as alternative therapies to medication, with fewer adverse effects and home-based use for tDCS. The ethical and societal impact of the abuse of NIBS for non-clinical use must be considered for policy-making and regulation implementations. This study adds to the neuroethical debate on the responsible use and application of NIBS technologies, taking into consideration the different perspectives of important stakeholders in the field.

## Introduction

Over the last years, the application of non-invasive brain stimulation (NIBS) methods, particularly transcranial direct current stimulation (tDCS) and repetitive transcranial magnetic stimulation (rTMS), has yielded great potential in the treatment of multiple neurological and psychiatric conditions, from chronic pain, cognitive impairments, to depression^1–5^. Despite the approval of rTMS as an effective treatment for major depression disorder across the globe^6,7^, the treatment is not reimbursed by most health insurances in many European countries, including Germany^8^. To minimize potential risks associated with NIBS use for research and the clinic, well-established and carefully considered safety rules for rTMS^4,7,9,10^ and tDCS^3,11,12^ have been developed.

In parallel, an increasingly financially lucrative market for tDCS devices for laypersons and the do-it-yourself (DIY) community, who use the technology mainly for cognitive enhancement, flourished in recent years^13,14^. Caputron (New York, USA), the biggest seller for direct-to-consumer tDCS devices, has grown 60–70% per quarter since its foundation in 2014, now selling tens of thousands of items per year^15^. Despite the evidence-based recommendations for the repeated application of anodal tDCS for patients with depression and fibromyalgia^3,5,16^, many direct-to-consumer tDCS devices for home use without intended use or adequate certification have no proven effectiveness and safety^17^ and various providers often advertise misleading promises^18^.

The rise of neurostimulation poses ethical considerations and concerns about its consequences on society as discussed in previous publications^19,20^. The application of NIBS for cognitive neuroenhancement in non-diseased groups has been critically discussed^21–23^ and calls for a broader and better policy approach to neurostimulation^24,25^ have highlighted the role of the neuroscientific community in policy making^26^ and the involvement of society and scientists in ethical debates^27^. Moreover, the inadequate regulation of devices for home use^15,28^ and oversight of direct-to-consumers neurotechnologies are frequently criticized^29^. Special consideration should furthermore be given to different perspectives on NIBS and the reduction of conflicts of interest^30^.

The development and use of neurotechnologies usually involve a set of diverse and often conflicting ethical imperatives^31^. Patients may want easily accessible and usable home stimulation devices, but regulators fear the consequences of potential abuse and misuse of these technologies. These discrepancies among stakeholder groups can be addressed through participatory processes, which include all potentially involved and affected stakeholders, such as individuals from different disciplines involved in the planning and production of devices. This includes commissioners and collaborators, users of NIBS and individuals who can offer their opinions and experiences to help shape the use of development of the technology^32–35^. To successfully include individual perspectives, participants must move from being passive respondents to becoming active creative producers, which can be achieved through co-creation^36^. Design-based workshop methods are ideal to enable a deep engagement with the topic, such as using speculative objects, which are meaningless on their own but serve the participants as a projection surface for their own thoughts.

Through tailored design-based and co-creative workshops, in line with Wexler and Sullivan^37^, our study included seven different stakeholder groups with personal experience or professional expertise in NIBS to share their perspectives as ‘experts of their experiences’^38^. Our main objective was to integrate different perspectives on the topic of NIBS, to highlight the heterogeneity of perspectives in the field, and enable derivations into practice by formulating concerns and wishes. This comprehensive and practice-oriented work also aims to outline the different aspects to consider for future developments in the field of NIBS.

## Methods

### Study design and procedure

This qualitative study was part of the Ethical, Legal, and Social Aspects (ELSA) of Neuroscience 2020 call, funded by ERA-NET Network of European Funding for Neuroscience Research. It started with a literature review to assess the latest developments and the current state of the science in NIBS, used for the classification of topics in workshops. The study comprised 9 tailored co-creative workshops to 7 different stakeholder groups; (1) patients who received tDCS or rTMS interventions, (2) students with basic knowledge and experience with NIBS, (3) clinical practitioners who stimulated patients for outpatient care or within a clinical trial, (4) DIY home users who were using tDCS devices bought online or self-built devices without medical prescription or supervision, (5) NIBS industry representatives, (6) philosophers and (7) policy experts, to evaluate the current developments in NIBS and their respective hopes and concerns on the matter. The qualitative data was anonymously collected and used to summarize perspectives from the different groups, and the study was carried out by a multinational team of authors to collect results representing more than one specific country. The workshops took place between June 2022 and January 2023 and were delivered by trained team members in a hybrid manner, with on-site meetings in Germany and Spain for patients, and online sessions for the other stakeholders via Microsoft Teams (Microsoft, Washington, USA) and the virtual whiteboard platform Conceptboard (Halle, Germany). All workshops were performed in accordance with the Declaration of Helsinki.

### Participants

We included 91 participants from 12 different countries pertaining to one of the previously defined stakeholder groups. Participants’ demographics, nationality, and experience with NIBS, self-reported via an online questionnaire, are summarized in Table 1. All the patients received repeated anodal tDCS over the primary motor cortex for the treatment of chronic pain. DIY-users were all patients suffering from mental disorders (n = 3) and chronic pain (n = 1) who bought a tDCS device online and administered the therapy by themselves without any medical prescription or supervision. The students from multidisciplinary fields had a basic knowledge of NIBS through their subjects. The industry representatives belonged to companies building tDCS and TMS devices for use in research and therapeutic use. Amongst the clinical practitioners, 29% worked with tDCS, 14% with TMS only, and 57% with both technologies. The sample for each group (e.g. more female participants suffered from chronic pain than males) was not gender-matched. Most of the participants in this study were from Germany (n = 42), followed by Spain (n = 35).

**Table 1:** Overview of characteristics of stakeholder groups, including number of workshops organized.

Stakeholder group	# of workshops organized	# of participants	Age (min–max, years)	Gender	Specification (# of respective group)	Nationality (# of respective group)
Patients	2	16	30–70	10 F, 6 M	Treatment of Chronic Pain: tDCS only (12), tDCS and rTMS (3), rTMS only (1)	Germany (6), Spain (10)
Students	2	34	20–35	24 F, 10 M	Students from: Neuroscience (11), Psychology (11), Medicine (7), Physics (3), Biomedical Engineering (2)	Germany (13), Spain (21)
DIY-user	1	4	20–59	0 F, 4 M	Usage for: self-treatment of unipolar depression and mental problems (3), self-treatment of chronic pain (1)	Czech (1), France (1), Norway (1), UK (1)
Clinical practitioner	1	7	30–69	4 F, 3 M	Treatment of routine patients (4) and within clinical trials (3) Professional background: Medical doctor (2), Psychologist (2), Medical Technical Assistant (1), Biologist (1), Ergotherapist (1)	Germany (7)
Industry representatives	1	5	29–55	1 F, 4 M	Working fields: Marketing (1), Sales (1), Research & Development (3)	Germany (1), UK (1), Spain (1), Italy (2)
Philosophers	1	11	20–60	5 F, 6 M	Experts from the fields of Philosophy (4) Neuroethics (3), Emerging Technologies (2), Technology and Science (1), Science Theory (1)	Germany (6), Taiwan (1), Switzerland (1), UK (1), Italy (1), Austria (1)
Policy experts	1	14	30–65	8 F, 6 M	Working at: State Agencies (6), Universities (8)	Germany (9), India (1), Spain (2), Australia (1), UK (1)

^#^, number; F, female; M, male; UK, United Kingdom.

Patients and students were recruited at the University Medical Center Göttingen in Germany and the National Hospital of Parapléjicos in Spain. Members of the other stakeholder groups were identified through research, e.g. by searching for companies and employees with relevant involvement with NIBS technologies, who were then invited by the research team. Participants were required to sign an informed consent form before participation. All participants were offered monetary compensation for participation, which was only accepted by students and DIY users.

### Co-creative and design-based workshop structure

**Fig. 1:**
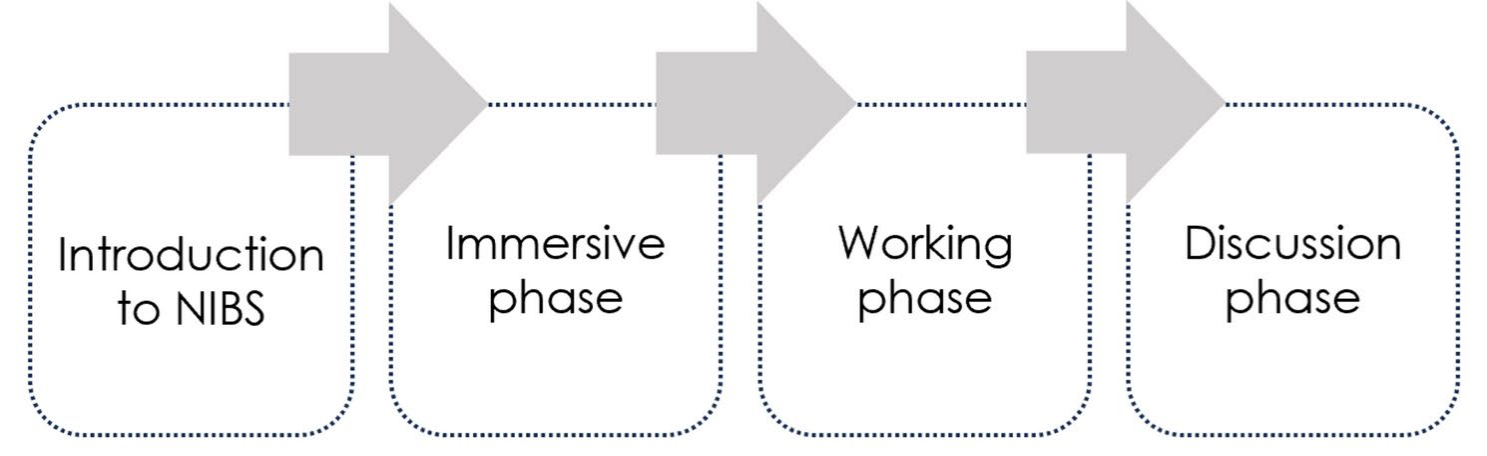
Design of tailored workshops illustrating the four phases. Introduction to NIBS: participants were informed about the basic physical and technical functions of NIBS, its application, and the current scientific effectiveness of the methods. Immersive phase: participants were guided to delve deeper into the topic of NIBS and to look at the topic from different perspectives while working co-creatively. The immersion methods as well as the methods and material used in the working phase were adapted for the different workshop groups. Working phase: participants individually or in small groups worked out their wishes and fears regarding NIBS using workshop material tailor-made for each stakeholder group. Discussion phase: each participant was invited to share their wishes, challenges and fears about NIBS, and the other participants could ask questions or make comments.

The general workshop procedure for each stakeholder group consisted of 4 phases, as illustrated in Fig. 1. In the on-site workshops, the working phase was assisted with workshop material, such as workbooks, sticky notes and speculative objects, while in the online workshops, this was done on the virtual whiteboard. Each workshop concluded with a discussion round. The details of the workshops for each stakeholder group are summarized below:

*Workshops for patients:* Two co-creative and design-based on-site identical workshops were each conducted at the University Medical Center Göttingen and the National Hospital of Paraplejicos. During the immersive phase of this workshop, participants were invited to go through the different steps of their treatment (How did they find the treatment option? How do they travel to the treatment facility? What happens during the treatment? What happens afterwards?). Then, they selected an associative object which represents fears, challenges and wishes regarding their treatment. Associative materials included a piece of rubber, a sponge, and a mirror, among others. After the selection of the associative material, the participants discussed the fears, challenges and wishes with the other patients and formulated wishes regarding the future of NIBS based on this discussion.

*Workshops for students:* Two identical virtual workshops were organized for students who used NIBS methods including tES and TMS during their academic career. During the immersive phase of the workshop, the groups were subdivided and exposed to different fields of application for NIBS- medical and psychotherapeutic use, performance enhancement in specific professions, and personal neuroenhancement. Afterwards, the participants were asked to create fictional newspaper articles, scientific articles, television series and biopics, based on the immersion. They presented them to the bigger group and discussed their fictional scenarios hinging on their wishes, hopes and fears regarding the technology. At the discussion phase of the workshop, they were asked about the needs and requirements for a wishable future with NIBS.

*Workshop for the DIY community:* One virtual workshop was organized for members of the DIY community. After the introduction to the topic of NIBS, everyone was asked to describe how and why they are using NIBS. Afterwards, they were asked to build their “ideal NIBS stimulation set-up” with the help of virtual stickers on the virtual whiteboard. They were asked to show what should happen ideally before, during and after the treatment. Afterwards, they presented their scenarios and discussed the discrepancies in reality and the steps to be taken to close the gap between the ideal scenario and reality.

*Workshop for industry representatives:* One virtual workshop was organized for industry representatives. After the introduction to neurotechnology, everyone was asked to describe their contact points to NIBS in their professional career. Afterwards, they were asked to build an “ideal NIBS stimulation device” with the help of a virtual sticker on a virtual whiteboard. They were then asked to present the device to the group and to discuss why it is nowadays not possible to have such a device and what would needed to have one in the future.

*Workshop for practitioners:* One virtual workshop was organized for medical practitioners who applied NIBS for the treatment of patients either as part of clinical trials or daily treatment routines. During the immersion phase, the participants were asked to develop a persona of a practitioner to answer the following questions: What does the persona like at her/his work? What not? What is the specific role of the persona in the treatment procedure, in the hospital, the health care system and society? Afterwards, they were asked to select a virtual sticker, which represented specific challenges and fears of the persona. In a final discussion, these stickers were presented to the group and potential ideas on how these challenges could be handled in future were developed.

*Workshop for philosophers:* One virtual workshop was organized for philosophers from the field of philosophy of mind and neuroethics. The participants were presented with a summary of all results from the previous workshops. The results were presented separately for the different topics. The participants were asked to choose one aspect which seems to be the most important to them and to describe which ethical or philosophical constructs could be underlying this or could influence an evaluation of these points.

*Workshop for policy experts:* One virtual workshop was organized for policy experts from the field of healthcare regulation. The participants were also presented with the results of previous stakeholder groups, similar to the philosopher group. Afterwards, they were asked to choose one potential impact identified by previous stakeholder groups and define which requirements are needed from a regulatory side to amplify or avoid this impact. In the final discussion, everyone presented the results and the other participants could comment on this.

### Analysis strategy

During each workshop, the participants’ verbal statements were recorded in writing by an experienced note-taker. In addition, the participants wrote down their key points on the analog or virtual worksheets. These results were compiled and inductively evaluated after each workshop, following Mayring^39^ such that there was a step-by-step formulation of the categories out of the materials. Statements were either assigned to existing categories or, if they did not fit anywhere, placed into new categories. In the first half of the evaluation, the categories created were checked for reliability and adjusted if necessary. At the end of the evaluation, a final reliability check was carried out and the individual categories were described with short summaries. A new, independent system of categories was developed for each workshop group. The MAXQDA 2022.0.0 software was used for the evaluation of the materials.

## Results

### Perspectives from patients

Patients’ perspectives on NIBS were categorized into (i) therapeutic application, (ii) efficacy, (iii) access and distribution, and (iv) recognition and information.

Most patients reported the challenging ergonomic conditions during rTMS or tDCS in the clinic, wishing for a *more comfortable and welcoming treatment room* for easier relaxation. Some patients complained about the use of rubber bands placed on the head during tDCS to fix the electrodes, which could be painful. Patients also emphasized the benefits of group tDCS interventions, ‘*a problem shared is a problem halved’,* and wished for personalized therapies.

The central wish of the group was symptom improvement to restore functional levels and alleviate symptomatology without taking pharmaceutical drugs. Major frustrations expressed by patients were the desire for longer-lasting therapeutic benefits and the difficulty *in coping with the disappointment that a non-functioning treatment can cause.*

Many patients wished that *health insurances, particularly public ones*, would *finance* the costs of NIBS therapies, including rTMS and tDCS. Daily travels for on-site stimulation were criticized, as it was difficult for patients to integrate the commuting into their routines, especially if they lived far away from the medical center. Thus, patients called for improved local accessibility and home-based tDCS under the supervision of trained professionals.

Moreover, participants wished for further information on the expectations, duration of therapeutic benefits, and follow-up treatments, especially during their first encounter with the technology. Patients with years of unsuccessful treatment attempts requested greater recognition during the intervention by being taken seriously and noticed.

### Perspectives from students

Students aspired for more efficient stimulation protocols optimized for specific conditions, which could lead to *reduced use of pharmaceutical treatments*- often accompanied by *undesirable secondary effects*. Further research was proposed to enable more efficient treatment, e.g. the ability *to stimulate deeper brain areas* and the development of *biomarkers to define responders to* rTMS and tDCS.

Discussing the use of NIBS to boost performance at work, participants expressed concern that the higher performance achieved with NIBS will become the new performance standard and that people who cannot afford and/or do not want the technology will be left behind. Nevertheless, NIBS could be useful in increasing working performance and accuracy, for instance for individuals working in the surgical field, in air traffic control, or on night shifts.

Students feared that many suppliers of stimulation devices for laypeople might enter the market with unsafe or ineffective devices in the future, raising concerns about aggressive and inaccurate marketing. They desired stricter regulation of NIBS to ensure that only effective and safe devices are on the market. Many students proposed that NIBS should only be allowed for use by professionally trained persons to avoid unintended incidents.

With the increased popularity of NIBS, more untrained people could *abuse the technology*, with potentially unpleasant consequences for society. The *risk for addiction and overuse* of NIBS remains a concern for some students. Furthermore, they noted the existing *gap between real life and laboratory experiments* about the exact interrelationships and effects of NIBS. Hence, the possibility of *unforeseen and unknown long-term effects* cannot be ignored.

### Perspectives from the DIY community

The different outlooks of home users were divided into (1) comfort, informed use, and supervised stimulation, (2) data protection, and (3) sharing personal experience.

A relaxed and comfortable environment, without the need for long-distance trips to professional practitioners, was a key reason for using home-based tDCS. Participants desired a stronger regulation of tDCS and perceived the use of unregulated devices from unknown suppliers as inappropriate. To feel safe, participants wished for in-person or virtual communication with healthcare professionals to receive adequate information on the home use of tDCS*.*

The participants saw the potential of networked devices but were disquieted by the potential risks of cyber hacking; ‘*What happens if a hacker finds a remote button and triggers my head?*’ Many participants coveted the virtual monitoring of tDCS via smartphone to choose the right stimulation protocol and to have a direct contact person in case of emergencies.

Participants considered the risk of side effects and addiction to be low, as compared to medication. They noted that innate biological differences in individuals are already unfairly distributed and that from this viewpoint, it would be fair to equalize these differences with the help of brain stimulation. For example, by equalizing differences in learning and concentration skills between individuals with the help of tDCS. Many users were concerned that privileged individuals would get easier access to neurotechnologies, exacerbating existing inequalities.

### Perspectives from industry representatives

The outcomes for industry representatives were categorized into (1) home use of tDCS, (2) standardization and research, (3) regulation of NIBS, and (4) public opinion.

Industry representatives applauded the benefits of home-based tDCS, which could cut down healthcare costs and improve accessibility to patients. Remote monitoring of the intervention via telemedicine by medical personnel was advocated for safer application. Participants also supported bigger investments in research to ensure the safety, and feasibility of home devices.

Participants highlighted the quintessence of open access to research findings and technological developments. They commented that restrictive patents should thus be avoided, as they represent obstacles along the path of standardization and broader application of NIBS methods, including rTMS and tDCS. Future integrations of the rapidly developing artificial intelligence (AI) and the vast data available could lead to larger investments by major players in the market, hence demanding anti-monopoly regulations. Furthermore, participants called for governmental funding for larger clinical trials and the promotion of start-up companies, alongside an exchange between research and industry.

Representatives called for the multiple ethical and regulatory barriers to be lifted. They denounced the intercountry differences in NIBS guidelines within the European Union (EU) and called for standardization. They also criticized the tedious and lengthy processes for ethical approvals of clinical studies, usually involving decision-makers with an inadequate understanding of NIBS and its application.

All of the representatives addressed the implications of non-clinical applications of the NIBS in the military, neuro-doping in sports, and neuroenhancement as this might further delay accessibility to patients. The participants understood that brain stimulation without background knowledge and experience within the field can seem daunting to the public. Therefore, there is a *need for more awareness within society regarding the importance of brain research*.

### Perspectives from medical practitioners

Participants desired compulsory certified training for the clinical use of NIBS, which should also be open to persons from non-medical fields such as scientists. Practitioners demand a curriculum that is constantly updated based on the latest research findings so that practitioners know how the individual treatment can be made as safe and effective as possible. Networking between practitioners should also be promoted within this framework.

To overcome the gap in knowledge about NIBS and its applications for users, participants conveyed a common desire for a centralized, trustworthy information site that details the mode of action, application, and risks neutrally and clearly, preferably provided and regulated by national health authorities. Due to the current strict data protection rules, it is often not possible to share stimulation data between research organizations, which could be improved through shared secured databases or coordinated consent forms.

Professionals involved in both clinical trials using NIBS and routine clinical work were discouraged by the heavy workload and stress from this frequent double burden. Working with rTMS was described as physically challenging due to uncomfortable holding positions of the coil for a significant duration. Participants aspired for a more ergonomic workplace and wished for more influence on therapy planning and decision-making for patients, which can mostly only be executed by medical doctors who might not be experts in the field of NIBS.

### Perspectives from philosophers

Philosophers expressed their thoughts on the partial evaluation of the technology based on fictional assumptions about a dystopian development, rather than its actual current capability. Instead, there *needs to be a distinction between thought experiments and the state of the art*. One philosopher pointed out that *there is a need for an epistemological position, an ethical stance, and a legal framework that can cope with persistent uncertainty* due to ongoing developments in the field of NIBS*.*

Participants raised the question of legal responsibility for NIBS use, for instance, in the case of harm caused by misuse, and proposed that the *distributions of responsibility, liability, and culpability must be included* in the thought process for agency. The philosophers noted that establishing a connection between neuronal data and the ‘self’ is a prerequisite to making statements about authorship and ownership of neural data.

Participants addressed their concerns on how non-reflected adoption of norms can lead to misconceptions, unnecessary pathologization, and stigmatization. The definition of *normal mental states* as well as *cultural and individual aspects* must also be included. The philosophers emphasized that neural data does not fit into any current data classifications, raising unanswered questions on data privacy and protection. They criticized the use of the term ‘non-invasive’, as such techniques like NIBS, though not physically invasive, could be mentally invasive, Participants proposed that it should be considered *whether mental invasiveness is morally relevant for clinical, research, and regulatory decision-making.*

Participants wished for the *definition of overuse, avoidance of stigmatization,* and differentiation between ‘good’ and ‘bad’ applications of the technology The philosophers demanded a rational regulatory framework, including *clear guidelines for access and control for patients and practitioners* and *the regulation of accessibility of NIBS for recreation use*. Fears were expressed regarding the uncontrolled use and the illegal trading of NIBS.

### Perspectives from policy experts

The policy experts noted the low motivation of manufacturers of NIBS technologies based on the consideration that the market might not be profitable. Participants recommended the inclusion of companies in the decision-making of NIBS regulations. European policy makers regarded the EU- and US-centricity as challenging since according perspectives tend to overlook intercultural differences in regulating and using NIBS (e.g. differences in the perspectives between individualistic and collectivistic cultures). To establish new regulations on NIBS, they proposed that the initial step must be taken at the level of the EU Commission, requiring a country to lead the new regulatory initiative. Laws are then needed at different levels from domestic law to soft regulation to governmental implementation to enforce regulations at the EU level.

Experts noted that the insufficient knowledge on the mechanisms of the techniques and lack of public awareness make it difficult to press legal bodies into drafting new policies on the therapeutic use of NIBS. They recommended a focus on the specific use of NIBS; for instance, the indication for depression treatment is mixed with lifestyle enhancement in healthy individuals. Similar to philosophers, the experts wished for clear definitions of which technologies are considered part of NIBS. Health insurances were referred to as gatekeepers to the technologies, with health insurance admission allowing broader accessibility to the technology. Participants also highlighted how NIBS technologies being seen as medical devices mandate specific regulations such as exemption for certain groups, consumer protection, and regulation for specific factors. Nevertheless, it was emphasized that evidence for therapeutic use must first be proven by comparative studies.

Participants highlighted the importance of research in law to identify existing policies and contact involved stakeholders to work out proposals for better regulation of NIBS, as well as testing the efficacy and safety of existing devices, with compulsory publications of results. They also proposed a research framework by installing study registries and databases for study findings in a specific timeframe following the completion of studies.

## Discussion

To our knowledge, this is the first inter-stakeholder perspectives study on NIBS, which included 7 stakeholder groups comprising patients, students, industry representatives, DIY home users, clinical practitioners, philosophers, and policy experts who participated in co-creative and design-based tailored workshops. The collected findings provide a good overview of which aspects are or could become important for NIBS. The inclusion and integration of perspectives of different concerned stakeholders can promote the faster implementation of neuroethical findings such as in the development of NIBS guidelines and recommendations to policy-makers, as compared to involving only one participatory group^37^.

Our findings hint towards tDCS and rTMS as promising tools for the treatment of multiple diseases with which many hopes are associated, as described^3,5,16^. Patients highlighted the importance of personalizing protocols and making the environment for on-site stimulation more ergonomic. There is a central desire across stakeholder groups (especially for patients and the DIY-community) to improve access to NIBS therapeutic methods to patients through the development of remotely controlled and supervised home-based tDCS applications and reimbursements by health insurers, in line with previous research^40–42^. Developing a neutral and trustworthy information site on treatment options, efficacy, and risks, controlled by an independent agency for medical practitioners, patients, and the public would be a way to prevent misconceptions and alarmism about NIBS.

However, the application of NIBS is not limited to clinical populations. The current and future implementations of NIBS in the military^43,44^, in sports^45,46^ and for neuroenhancement in healthy individuals^47^ should not be undermined^1^, which was also addressed in our study. While NIBS in the workplace could be beneficial, this could put implicit pressure on employees, as observed by the students. Neuroenhancement in the workplace could further lead to a coercive and abusive use of NIBS devices on workers in pursuit of productivity and potential negative consequences of neurological fatigue and stress^48,49^. This raises an ethical and moral debate on what determines the application of NIBS as ‘good’ (e.g. treatment of an illness) or ‘bad’ (e.g. performance enhancement). Until today, there is no consensus on the use of NIBS methods such as tDCS and rTMS regarding their moral status in society, with unanswered ethical questions, that can hinder the future application and development of the field^48–50^. Hence, it is essential to have a strict regulatory framework for the access to and use of NIBS devices.

For successful implementation of NIBS therapies, adequate training must be provided to clinical practitioners and secure sharing of stimulation data is a prerequisite for safely monitoring home-based interventions. Data sharing should also be viewed in the light of the unclear categorization of and ways to deal with neural data during NIBS, as pointed out by philosophers. The involvement of AI in the field of NIBS in the future is undeniable and will boost the development of NIBS technologies^51^. This could pose a risk of even greater monopoly formation, as the costly research and large amounts of data required for this are more likely to be provided by large market players.

The findings of this study were discussed further by a team of scientific experts through a rigorous impact assessment. The different perspectives were then discussed by an interdisciplinary panel of experts including neuroscientists, clinical neurophysiologists, psychiatrists, lawyers, ethicists, philosophers, and patients’ self-help group representatives to draft a set of participative developed recommendations for NIBS in the European Union, which was published as Maier et al.^52^.

### Methodological strengths and limitations

Accounting for the ethical challenges stemming from the use of emerging and rapidly developing NIBS technologies, a broad societal inclusion of stakeholder groups with diverse opinions on the matter in a participatory process has been shown to be successful in neuroethical deliberations^53–55^. Multifaceted perspectives can address important current issues associated with the available technology, assess its potential, and provide directives for the future of the field. Nevertheless, an inclusive participatory process can be challenging, bringing with itself the task of including stakeholders from different disciplines, like neuroscience, medicine, policymaking, and philosophy who are involved in the research, use, and assessment of NIBS, speak different languages and have different thought processes based on their field of expertise. In our study design, this was taken into consideration as we designed workshops tailored to each stakeholder group. To successfully combine the different types of knowledge and perspectives from the stakeholder groups, we ensured a diverse project team with different disciplines such as medicine, neuroscience, and ethics^56^ for an adequate and accurate ‘translation’ of the information from each stakeholder group to an inclusive language. The use of design-based methods, such as the application of associative speculative materials in the workshops for patients, in participative research can help to engage the individuals in discussion about the topic by simplifying complex and abstract contexts. Creative methods, as implemented in this study, make aspects of knowledge accessible that are difficult to verbalize^57^. Another strength of our protocol lies in considering the dimensions of the Quadruple Helix framework, proposed by Carayannis and Campbell^35^. This concept entails the inclusion of culture- and media-based society in research while emphasizing co-existence and co-development of diverse knowledge modes together with mutual learning between stakeholders. We encouraged the individual stakeholder groups to consider possible impacts on other groups, for instance, industry representatives commenting on the future medical implications of NIBS.

Our findings should be interpreted in the light of the study’s limitations. Despite the inclusion of seven different stakeholder groups, other groups might have also been relevant to the process, including representatives of health insurance companies, parents of children in need of treatment, digitalization and AI experts, or health sociologists. It can be critically discussed to what extent cultural differences play a role in the perception of NIBS. Despite the efforts to include individuals from non-European countries, the predominantly Eurocentric distribution of participants, with a majority from Spain and Germany, undermines the generalizability of the results. In this study, NIBS techniques were limited to tDCS and TMS. Future studies should include other methods such as transcranial ultrasound stimulation and transcranial static magnetic stimulation, among others, while assessing stakeholder considerations. The sample size in each stakeholder group was heterogeneous, ranging from four participants in the DIY group to 34 in the students’ group, and they were not gender-matched. The patient group consisted of only chronic pain patients treated with anodal tDCS over the primary motor cortex. Having larger group sizes and a more diverse patient group treated with different methods of NIBS should be considered for future participatory processes about brain stimulation involving stakeholder engagement.

## Conclusion

In summary, this study on NIBS brings together diverse stakeholder perspectives, shedding light on crucial considerations for its development and implementation. The findings underscore the potential of NIBS, in treating various conditions, while emphasizing the need for personalized protocols and improved accessibility. However, ethical dilemmas arise regarding its broader applications, including military, sports, and workplace enhancement, necessitating a robust regulatory framework. The study’s methodological strengths, such as inclusive participatory processes and interdisciplinary collaboration, offer valuable insights but are accompanied by limitations, including sample heterogeneity and Eurocentric bias. Moving forward, efforts to broaden stakeholder inclusion, expand research to encompass diverse NIBS techniques, and address ethical concerns are essential for the responsible advancement of this field.

## Data Availability

The datasets generated and analysed during the current study are not publicly available in order to guarantee the anonymity of the participant but are available from the corresponding author on reasonable request.
